# Dihydromyricetin improves vascular hyporesponsiveness in experimental sepsis via attenuating the over-excited MaxiK and K_ATP_ channels

**DOI:** 10.1080/13880209.2018.1478430

**Published:** 2018-07-13

**Authors:** Jin Peng, Jian Zhang, Li Zhang, Yonggang Tian, Yahong Li, Lujun Qiao

**Affiliations:** Department of ICU, Shengli Oilfield Central Hospital, Dongying, China

**Keywords:** Ampelopsis grossedentata, vasoplegia, vascular hyporeactivity

## Abstract

**Context:** Dihydromyricetin (DMY) has oxidation resistance, anti-inflammatory and free radical scavenging capabilities. The preventive effects of DMY for vascular hyporeactivity remain unclear.

**Objective:** This study investigates the preventive effects of DMY in vascular hyporeactivity.

**Materials and methods:** The experimental sepsis was induced by transvenous administration of lipopolysaccharide (LPS) to Sprague–Dawley (SD) rats. DMY-treated rats received daily administration of DMY, 5 μg/kg dissolved in DMSO through the tail vein for 7 days. The invasive mean arterial pressure (MAP) of the caudal ventral artery was measured. Dose-response curves for norepinephrine (NE, doses from 10^−9^ to 10^−6 ^M) were obtained in isolated thoracic aorta in a cumulative manner. The function of MaxiK and K_ATP_ channels were investigated using whole-cell patch clamp recording. The Elisa was adopted to measure the serum concentration of NO, MDA, 3-NT, IL-1β and TNF-α.

**Results:** The increased MAP in septic rats induced by vasopressor agents was smaller than that in control rats. However, the % of increased MAP induced by vasopressor agents was raised by DMY injection (NE: 20.4 ± 8.495 vs. 15.16 ± 5.195%; AVP: 14.05 ± 2.459 vs. 9.583 ± 2.982%, *p* < 0.05). The vascular hyporesponsiveness to NE (10^−6 ^M) *in vitro.* was increased by 51% in LPS + DMY group compared with that in LPS + Con group (2.74 ± 0.81 vs. 1.82 ± 0.92 g, *p* < 0.05). Charybdotoxin (a potent MaxiK channel blocker) and glibenclamide (a K_ATP_ channel blocker) pretreatment, instead of 4-aminopyridine (4-AP) and BaCl_2_, could diminish the DMY-induced improvement of vasoconstrictor hyporeactivity (ChTX: 73.2 ± 11.8 vs. 71.8 ± 13.5%; Glib: 63.1 ± 12.5 vs. 58.1 ± 13.7%, *p* > 0.05). DMY blunted the highly sensitized MaxiK and K_ATP_ channels of arterial smooth muscle cells isolated from the thoracic aorta of LPS rats. DMY decreased the serum level of NO, MDA, IL-1β and TNF-α, which had increased in LPS rats.

**Discussion and conclusions:** Our results indicate that DMY administration ameliorated the impaired contractility of the rat aorta in experimental sepsis. Such an effect is mediated by normalization of the over-excited MaxiK and K_ATP_, channels possibly via oxidative stress inhibition.

## Introduction

Sepsis, often presenting with multiple organ dysfunction syndrome and organ failure, is a major problem that causes the most challenging efforts in intensive care today (Johnson and Mayers 2001). One of the most important clinical characteristics of sepsis and septic shock is the vascular hyporesponsivity to vasopressor agents (Donaldson and Myers 1996; Strunk et al. 2001). It represents an important condition for patient survival. Different therapeutic strategies aim to improve vital organ function (Leone and Martin 2008). The identification of new intracellular signalling related to sepsis progression might contribute to the development of therapeutic strategies to reduce sepsis-associated mortality.

The exact mechanism underlying the susceptibility of hypotension in patients with sepsis remains unclear. However, oxidative stress is associated with impaired vasoconstriction in sepsis (Szabo et al. 1995; Wu et al. 2004). According to Wu et al. (2004), antioxidant treatment, before cecal ligation and puncture (CLP) surgery to induce sepsis, increases mice survival and decreases hypotension, plasma NO metabolites, oxidative stress, NOS2 mRNA and angiotensin II (AngII) hyporesponsivity. In regard to endothelial cells, Huang et al. (2016) showed that lipopolysaccharide (LPS) treatment of human umbilical vein endothelial cells increases oxidative stress, malondialdehyde levels, superoxide dismutase 2 protein expression and phosphorylation of c-Jun N-terminal kinases, and decreases SOD1 expression. Furthermore, potassium channels in vascular smooth muscle cells (VSMCs), which are widely distributed in vasculatures, are reported to play an important role in the vascular tone regulation under pathophysiological conditions. Using patch-clamp techniques, Dopico et al. (2002) found that administration of large-conductance Ca^2+^-activated K^+^ (BK_Ca_, MaxiK, Slo1) channel blockers could eliminate vasodilation. Ample evidence from experimental animal models and the identification of K_ATP_ channel mutations in patients also indicate that K_ATP_ channel plays a critical role in vascular tone regulation (Shi et al. 2012).

Dihydromyricetin (DMY), a flavonoid compound, is extracted from the stems and leaves of *Ampelopsis grossedentata* (Hand.-Mazz.) W.T. Wang (Vitaceae). *Ampelopsis* is widely distributed in tropical and subtropical regions of the world. It is used in Chinese traditional medicine for treating liver disorders (Pang et al. 2011; Liu et al. 2017). Previous studies documented that DMY has oxidation resistance, anti-inflammatory, free radical scavenging capabilities among other biological activities (Zhang et al. 2003). The effects of DMY on LPS-induced vascular hyporesponsivity to vasopressor agents, has not been reported. This study explores the effects of DMY on vascular hyporesponsivity in an experimental sepsis model by focusing special attention on the role of VSMCs MaxiK and K_ATP_ channels in freshly isolated rat thoracic aorta.

## Materials and methods

### Animals

A total of 72 pathogen-free, adult male Sprague–Dawley rats (weighing 200–250 g) were obtained from the Shanghai Slac Experimental Animal Centre (Shanghai, China). The rats were housed in individual cages in a temperature-controlled room with alternating 12 h light/dark cycles. Food was withheld 8 h before the start of experiments, but all animals had free access to water. The study was approved by the Animal Care Committee of the Binzhou Medical College and performed in accordance with the Guide for the Care and Use of Laboratory Animals.

### Experimental design and sample collection

The experimental animals were randomly divided into four groups of 18 rats each: normal saline-injected rats treated with DMSO group as control (Con + NS), LPS-injected rats treated with DMSO group (LPS + NS), control rats treated with DMY (Sigma-Aldrich, St. Louis, MO), group (Con + DMY) and LPS rats treated with DMY group (LPS + DMY). DMY-treated rats received daily administration of DMY, 5 μg/kg dissolved in DMSO through the tail vein for 7 days. On the 7th day, rats in the DMSO and DMY groups were injected with 10 mg/kg LPS (Sigma-Aldrich, St. Louis, MO) through the tail vein. After 24 h, the animals were sacrificed and blood samples were transferred to tubes and immediately centrifuged (3000 rpm for 10 min at 4 °C). Serum samples were frozen and stored at −80 °C for biochemical analyses.

### In vivo measurement of the mean arterial pressure

On the 8th day, catheters for invasive arterial blood pressure monitoring were inserted into the caudal ventral artery. First, the rat was placed in the supine position, and the tail skin was sterilized with povidone-iodine. Then, a 24-gauge catheter (Terumo, Tokyo, Japan) was aseptically inserted into the caudal ventral artery to allow blood pressure measurement. After backflow from the catheter was confirmed, the catheter was connected to the introducer of the arterial line. Visualization of the arterial waveform on the monitor was confirmed, and the catheter was flushed with heparinized physiological saline.

### Artery isolation and in vitro vascular reactivity protocol

Thoracic aortas were isolated and prepared for vascular function studies (Spradley et al. 2012). On the 8th day, rats were anesthetized using 300 mg/kg chloral hydrate and decapitated. The thoracic aorta was carefully excised and placed in a Petri dish filled with cold Kerbs solution (KHS) containing (in mM) NaCl 118.5, KCl 4.7, KH_2_PO_4_ 1.2, MgSO_4_ 1.2, NaHCO_3_ 25.0, CaCl_2_ 2.5 and glucose 5.5 at 37 °C continuously bubbled with a 95% O_2_ to 5% CO_2_ mixture (pH 7.4). The aorta was cleaned of excess connective tissue and cut into rings of approximately 3 mm in length. Thoracic aorta segments were mounted on two parallel stainless-steel pins for arterial isometric tension recording through a MAP2000 isometric force transducer (Alcott Biotech Co. Ltd., Shanghai, China) connected to a computer. Segments were suspended in an organ bath containing 20 mL of KHS and subjected to a tension of 2 *g* which was readjusted every 30 min during a 120 min equilibration period before drug administration. The vessels were then exposed to KCl (60 mM) to check their functional integrity. After washing out the thoracic aorta rings with KHS solution, we recorded the basal vascular tone prior to evaluating the contractile response by measuring the maximal peak height which is expressed as the maximal tension % achieved in response to 140 mM K^+^ (Kmax). Dose-response curves for NE (doses from 10^−9^ to 10^−6 ^M) were obtained in aortic rings in a cumulative manner. To explore the role of K^+^ channels in vascular tension, the contractility was quantitated after administration of 3 × 10^−3 ^M TEA (tetraethylammounium, a nonselective potassium channel blocker), 3 × 10^−8 ^M charybdotoxin (ChTX, a potent MaxiK channel blocker), 3 × 10^−8 ^M glibenclamide (Glib, K_ATP_ channel blocker) and 3 × 10^−3 ^M 4-AP (4-aminopyridine, a potent Kv channel blocker) and 3 × 10^−3 ^M BaCl_2_ (a potent Kir channel blocker).

### Aortic smooth muscle cells (ASMCs) isolation and electrophysiological recording

ASMCs were isolated from the rat thoracic aorta by enzymatic digestion. Growth of passage 1–2 ASMCs was arrested for 24 h in serum-free Dulbecco’s modified Eagle’s medium before electrophysiological experiments. Single cells were released using a fire-polished pipette and allowed to adhere to the bottom of a recording chamber (0.5 mL). All operations were performed at room temperature (20–25 °C). Whole cell patch clamp recording was carried out with an Axopatch 700B amplifier (Axon Instruments, SV, San Francisco, USA). The potential of the membrane was clamped at -60 mV, subsequently digitized at 10–50 kHz (Digidata 1440 A interface, Axon Instruments). The resistance of patch electrodes was 4–5 MΩ. The bath solution contained (in mM): 134 NaCl, 6 KCl, 1 MgCl_2_, 1.8 CaCl_2_, 10 glucose and 10 HEPES (pH 7.4). The pipette solution contained the following (in mM): 107 KCl, 1.0 MgCl_2_, 1.9 CaCl_2_, 10 HEPES, 5 EGTA, 25 KOH, 0.1 Na_2_ATP, 0.1 NaADP and 0.1 LiGTP (pH 7.2 adjusted with KOH, free Ca^2+^∼100 nM).

Transient outward BK_Ca_ currents were measured. A pre-pulse (0 mV, 100 ms) was followed by test pulses (400 ms) from −80 to +80 mV in 10 mV increments. Test solutions bathing the cytoplasmic face of the patch membrane contained (in mM): 145 N-methyl-d-glucamine (NMDG), 3 KCl, 0.6 MgCl_2_·6H_2_O, 2.5 CaCl_2_·2H_2_O, 10 HEPES, 10 glucose (pH adjusted to 7.4 with Tris-base and 300 mOsM). ChTX (100 nM) was added to extracellular solutions to isolate the ChTX sensitive BK_Ca_ currents (the control currents subtracted the ChTX non-sensitive currents).

Freshly isolated VSMCs were obtained for the recording of K_ATP_ currents at room temperature. With references to Koide et al. (2014), K_ATP_ currents were recorded at −60 mV. After a stable baseline was obtained, the extracellular solution was changed to 140 mM K^+^ solution, and the ‘140 mM K^+^’ bath solution was made by iso-osmotic replacement of NaCl with KCl. Pinacidil (10 μM) and glibenclamide (10 μM) were routinely added to extracellular solutions, pinacidil was used to increase the inward current, otherwise glibenclamide was used to inhibit the inward current, then the glibenclamide sensitive K_ATP_ currents were recorded.

### Measurement of NO, MDA, 3-NT, IL-1β and TNF-α in serum

The Elisa reagents kits were purchased from Jiancheng Biologic Company (Nanjing, China). The method for plasma nitrite and nitrate levels (as a measure of NO) was based on the Griess reaction. Total nitrite was measured by spectrophotometry at 545 nm after conversion of nitrate to nitrite by copperized cadmium granules. MDA, the oxidative stress (OS) product of lipid peroxidation reacts with thiobarbituric acid under acidic conditions at 95 °C to form a pink-colour complex with an absorbance at 532 nm. Rat serum 3-nitrotyrosine (3-NT), the OS product of proteins and the proinflammatory cytokines (TNF-α and IL-1β) levels were measured, using a microplate reader (Thermo Multiskan MK3) at 450 nm.

### Statistical analysis

Quantitative data are presented as mean ± SEM. Statistical analysis was performed using SPSS (version 15, SPSS Inc., Chicago, IL) software. The basal vascular tension and serum ROS level in the control, LPS and LPS + DMY groups were analyzed using one-way ANOVA after significance is verified by the Dunnett’s multiple comparison tests. Then, a two-way repeated measures ANOVA was used to compare the response to different doses of NE, with again Holm-Bonferroni *post hoc* tests if a significant difference emerged. Independent-sample *t*-test was used to assess differences between LPS and LPS + DMY groups. *p < 0.05 was considered statistically significant.

## Results

Animals in Con + NS, Con + DMY, LPS + NS and LPS + DMY groups did not show any alteration in their general status under the LPS and DMY injection conditions. On the 7th day after the surgery, there was no significant difference in the mean weight of the rats among Con + NS, Con + DMY, LPS + NS and LPS + DMY groups.

### DMY administration ameliorated LPS-induced vascular hyporesponsiveness in vivo

**Figure 1.**
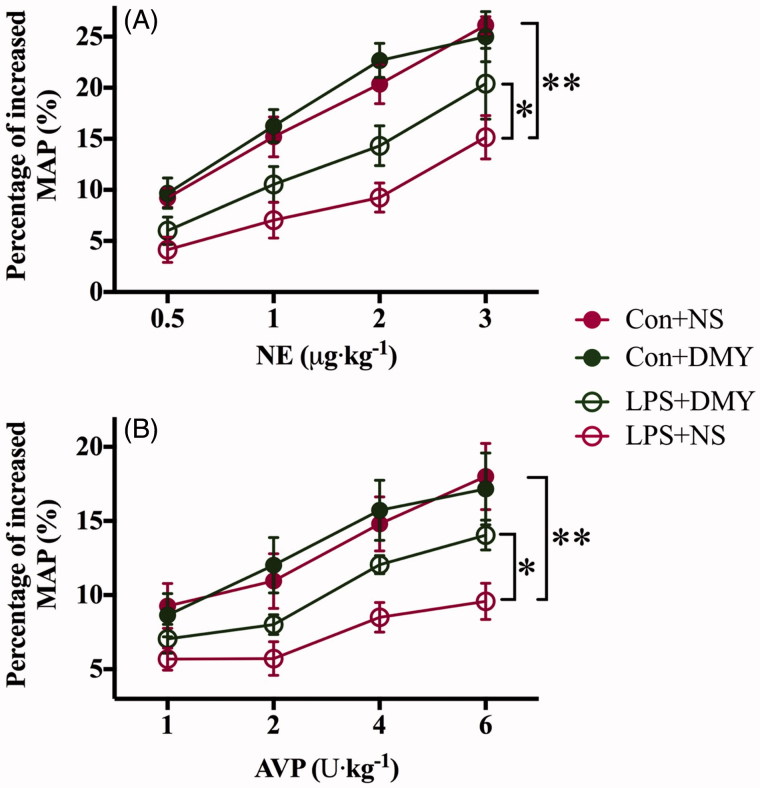
Dihydromyricetin significantly ameliorated the vascular hyporesponsiveness induced by LPS. (A) The % of increasing mean arterial pressure (MAP) of control and LPS mice with or without dihydromyricetin after different doses of norepinephrine (NE) administered (0.5, 1, 2, 3 μg/kg, *n* = 6). (B) The percentage of increase in MAP of control and LPS rats with or without dihydromyricetin after different doses of arginine vasopressin (AVP) administered (1, 2, 4, 6 U/kg, *n* = 6). The percentage of increase in mean arterial pressure: (increased MAP – basal MAP)/basal MAP. Data are presented as mean ± SEM. A two-way repeated measures ANOVA was used, with again Holm-Bonferroni *post hoc* tests if a significant difference emerged. *represents *p* < 0.05, **p < 0.01.

The percentage increase in mean arterial pressure (MAP) after NE or AVP administration in LPS + NS rats was much lower than that in Con + NS rats (3 μg/kg NE: 15.2 ± 5.2 vs. 26.1 ± 2.1%, *p* < 0.05, Figure 1(A); 3 U/kg AVP: 9.6 ± 3.0 vs. 18.0 ± 5.5%, *p* < 0.05, Figure 1(B)). The percentage increase in MAP induced by vasopressor agents was raised by DMY injection (LPS + DMY vs. LPS + NS, NE: 20.4 ± 8.5 vs. 15.2 ± 5.2%, *p* < 0.05, Figure 1(A); AVP: 14.1 ± 2.5 vs. 9.6 ± 3.0%, *p* < 0.05, Figure 1(B)). There was no significant difference between the Con + NS and Con + DMY group (NE: 26.1 ± 2.1 vs. 25.0 ± 6.0%, *p* < 0.05, Figure 1(A); AVP: 18.0 ± 5.5 vs. 17.2 ± 6.0%, *p* < 0.05, Figure 1(B)).

### MaxiK and K_ATP_ channels mediated the DMY-induced improvement of vasoconstrictor hyporeactivity in experimental sepsis

**Figure 2.**
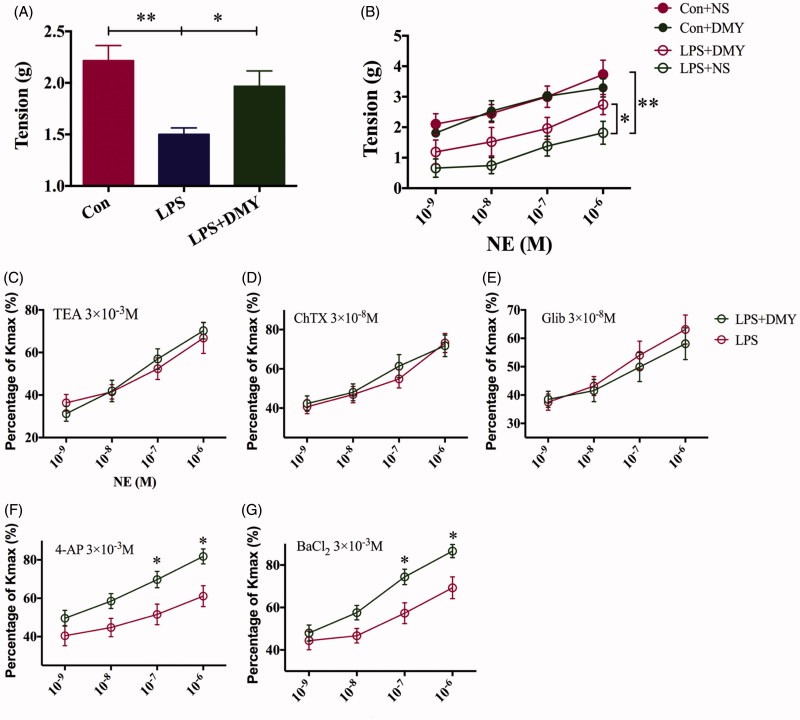
MaxiK and K_ATP_ channels in vascular smooth muscle cells played a critical role in vasoconstrictor hyporeactivity of LPS rats. (A) The basal vascular tone of thoracic aorta rings isolated from control, LPS and LPS + DMY groups (*n* = 6). The data were analyzed by using one-way ANOVA if significant by Dunnett’s multiple comparison tests. (B) The contractile response for arterial strips from control and LPS rats with or without DMY to norepinephrine (3 × 10 − 5 M, *n* = 6). (C) NE contractions with TEA pretreatment were expressed as % of maximal contraction elicited with K in LPS and LPS + DMY rats (*n* = 6). (D) NE contractions with ChTX pretreatment were expressed as % of maximal contraction elicited with K in LPS and LPS + DMY rats (*n* = 6). (E) NE contractions with Glib pretreatment were expressed as % of maximal contraction elicited with K in LPS and LPS + DMY rats (*n* = 6). (F) NE contractions with 4-AP pretreatment were expressed as % of maximal contraction elicited with K in LPS and LPS + DMY rats (*n* = 6). (G) NE contractions with BaCI_2_ pretreatment were expressed as % of maximal contraction elicited with K in LPS and LPS + DMY rats (*n* = 6). Data were presented as mean ± SEM. A two-way repeated measures ANOVA was used, with again Holm-Bonferroni *post hoc* tests if a significant difference emerged. *represents *p* < 0.05, **p < 0.01.

The basal vascular tone of the thoracic aorta rings isolated from the rats with LPS administration decreased markedly, however, DMY could reverse it (Control: 2.2 ± 0.4 g; LPS: 1.5 ± 0.2 g; LPS + DMY: 2.0 ± 0.4 g, Figure 2(A)). The contractile response for arterial strips to NE (3 × 10^−6 ^M) in LPS + NS rats was significantly blunted compared with that in Con + NS rats (NE: 1.8 ± 0.9 vs. 3.7 ± 1.1 g, *p* < 0.05, Figure 2(B); AVP: 4.1 ± 0.3 vs. 3.9 ± 0.1 g, *p* < 0.05, Figure 2(B)). The vascular hyporesponsiveness to vasopressor agents was improved by DMY injection (LPS + DMY vs. LPS + NS, NE: 2.7 ± 0.8 vs. 1.8 ± 0.9 g, *p* < 0.05, Figure 2(B)).

NE induced contraction divided by potassium-induced maximum contraction (Kmax) (NE-induce contraction/Kmax) was adopted to indicate the shrink ability of the isolated arteries. To explore which potassium channels played a pivotal role in DMY-induced improvements of vascular hyporeactivity, isolated arteries were pretreated with potassium channel blockers previous to NE incubation. After pretreatment of TEA (tetraethylammounium, a nonselective potassium channel blocker, 3 × 10^−3 ^M), the improvement of vasoconstrictor hyporeactivity induced by DMY was significantly blocked (10^−6 ^M NE: LPS + NS 70.3 ± 9.6 vs. LPS + DMY 66.8 ± 17.7%, Figure 2(C)). Meanwhile, the contribution of MaxiK, K_ATP_, Kv and Kir channels to DMY induced amelioration of vasoconstrictor hyporeactivity was examined. ChTX (a specific MaxiK channel blocker) and Glib (a specific K_ATP_ channel blocker) pretreatment could also diminish the DMY-induced improvement of vasoconstrictor hyporeactivity (ChTX: LPS + NS 73.2 ± 11.8 vs. LPS + DMY 71.8 ± 13.5%; Glib: LPS + NS 63.1 ± 12.5 vs. LPS + DMY 58.1 ± 13.7%, Figure 2(D,E)), while the same effect was not observed in 4-AP (4-aminopyridine, a specific Kv channel blocker) and BaCl_2_ pretreatment (barium chloride, a specific Kir channel blocker) used (4-AP: LPS + NS 81.8 ± 9.6 vs. LPS + DMY 61.1 ± 13.2%; BaCl_2_: LPS + NS 86.6 ± 7.6 vs. LPS + DMY 69.3 ± 12.6%, Figure 2(F,G)).

### DMY blunted the high sensitized MaxiK and K_ATP_ channels of ASMCs isolated from the thoracic aorta of LPS rats

**Figure 3.**
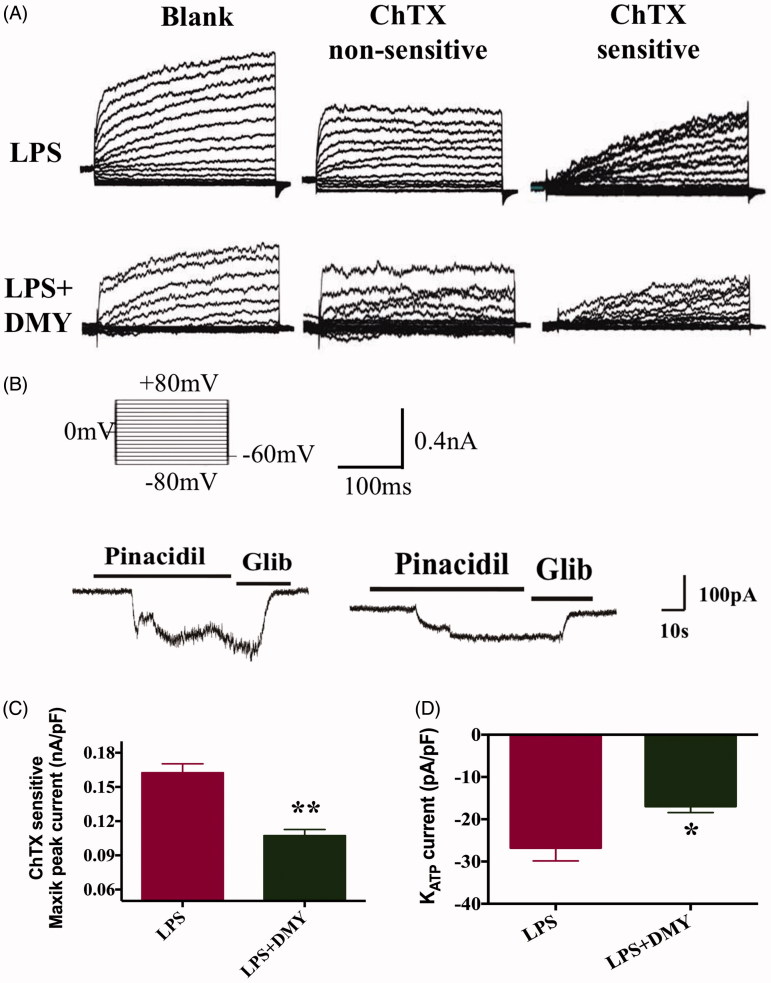
MaxiK and K_ATP_ currents in ASMCs from LPS and LPS + DMY rats. The whole-cell K^+^ currents in aortic myocytes measured by conventional whole-cell patch-clamp technique. (A and C) Typical recordings of MaxiK currents in ASMCs from LPS and LPS + DMY rats, respectively. In the group of LPS + DMY, BK_Ca_ peak currents were significantly smaller at the voltage −60 mV, compared with those in LPS group (*n* = 8, *p < 0.05). (B) K_ATP_ currents of ASMCs from LPS and LPS + DMY rats. 10 μM pinacidil was administered to increase an inward current, and then it was reverted by glibenclamide to obtain pinacidil-induced K_ATP_ currents in ASMCs from both LPS and LPS + DMY models. (D) The magnitude of K_ATP_ current in ASMCs from LPS + DMY rats was significantly smaller than that from LPS rats (*n* = 6, *p < 0.05). Data are presented as mean ± SEM. Independent-sample *t*-test was used to assess differences between LPS and LPS + DMY groups.

ASMCs were isolated from the thoracic aorta by using enzymatic digestion to obtain highly purified acute isolated ASMCs. Figure 3(A) illustrated that under the same experimental conditions, the whole-cell MaxiK currents density in LPS + DMY was smaller than that in LPS rats. The statistical analysis was shown in Figure 3(C) (0.2 ± 0.02 vs. 0.1 ± 0.02 nA/pF, *p* < 0.01). The whole cell recordings were carried out in a symmetrical 140 mM K^+^ solution to optimize the recordings, and the cells were held at a holding potential of −60 mV. Raising the extracellular K^+^ to 140 mM induced small K_ATP_ currents. Pinacidil (10 μM) was applied to increase an inward current in cells from LPS and LPS + DMY rats for enhancing the K_ATP_ currents (Figure 3(B)) and glibenclamide (a K_ATP_ channel inhibitor) could revert it in both cell types. Then pinacidil-induced K_ATP_ currents in isolated ASMCs from both LPS and LPS + DMY models were obtained. The magnitude of the K_ATP_ current in ASMCs from LPS + DMY rats was significantly lower than those from LPS rats (−26.7 ± 3.0 vs. −16.9 ± 3.7 pA/pF, Figure 3(D), *p* < 0.05).

### DMY decreased the serum concentrations of cytokines and oxidative stress increased by LPS injection

**Figure 4.**
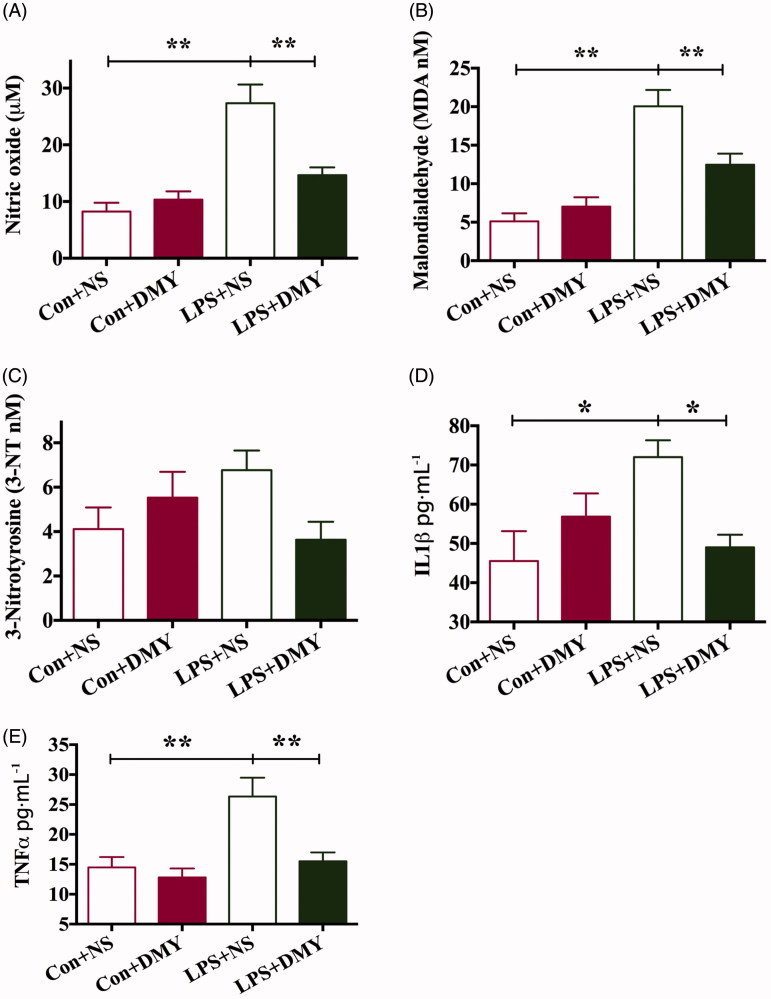
Levels of NO, MDA, 3-NT, IL-1β and TNF-α in serum from the control group, CON + DMY group, LPS + NS group and LPS + DMY group (*n* = 6). Data are presented as mean ± SEM. The data were analyzed by using one-way ANOVA if significant by Dunnett’s multiple comparison tests. *p < 0.05, **p < 0.01.

The serum level of NO, MDA, IL-1β and TNF-α significantly increased in LPS + NS rats compared with those in control rats (NO: 25.8 ± 3.6 vs. 8.6 ± 1.9; MDA: 20.1 ± 2.1 vs. 5.1 ± 1.0; IL-1β: 68.5 ± 6.0 vs. 45.5 ± 7.7; TNF-α: 26.3 ± 3.1 vs. 14.5 ± 1.7). However, the serum level of ROS was significantly reversed by DMY (NO: 14.9 ± 1.6; MDA: 12.5 ± 1.4; IL-1β: 50.7 ± 4.5; TNF-α: 15.5 ± 1.5). There was no significant difference in the serum level of 3-NT among the Con + NS, Con + DMY, LPS + NS and LPS + DMY (Figure 4).

## Discussion

The principal findings of this work were that DMY administration improved vascular hyporesponsiveness in LPS induced sepsis, via attenuating the over-excited MaxiK and K_ATP_ channels and free radical scavenging, this is supported by the following observations: (1) DMY administration ameliorated LPS-induced vascular hyporesponsiveness, represented by increased MAP; (2) MaxiK and K_ATP_ channels mediated the DMY produced improvement of vasoconstrictor hyporeactivity in experimental sepsis; (3) DMY blunted the highly sensitized MaxiK and K_ATP_ channels of ASMCs isolated from the thoracic aorta of LPS rats; (4) DMY decreased the degree of oxidative stress induced by LPS injection. Taken together, we verified for the very first time that MaxiK and K_ATP_ channels mediated the improvements of vascular hyporesponsiveness in LPS injected rats.

It has been reported that DMY exhibited antioxidant capacity via increasing the activity of heme oxygenase-1 (HO-1) (Kou et al. 2012) and the acid-fast activities of DMY were mainly attributed to its *ortho*-trihydroxy group (Xin et al. 2012). A previous study suggested that DMY inhibited T cell activation and secretion of related cytokines by binding to the 46th cysteine of IKKβ and inhibiting IKKβ kinase activity (Xin et al. 2012). The important immune cells, macrophages and T cells, play a pivotal role in oxidative stress of septic shock. Macrophages are not only involved in the inflammatory response, but also participate in the secretion of inflammatory factors COX-2, iNOS, TNF-α and IL-1β (Won et al. 2006). It has also been reported that DMY can inhibit the release of iNOS, IL-6 and other inflammatory factors. This effect can be attributed to inhibition of IKKβ, IKBα and NF-κB signal transduction pathways (Hou et al. 2015). In this study, we observed the preventive effects of DMY on LPS-induced vascular hyporesponsiveness in rats. The results showed that DMY could significantly relieve vascular hyporesponsiveness caused by LPS and this effect could be blocked by MaxiK and K_ATP_ inhibitors incubation previous to NE treatment, which suggests that DMY may inhibit the inflammatory response, thus reverse the over-excited MaxiK and K_ATP_ channels in VSMCs.

Accumulating evidence shows that the activity of ion channels, especially the potassium channel on ASMCs, plays a pivotal role in affecting the contractile state of the peripheral arteries. Excess opening of the potassium channel in ASMCs caused membrane hyperpolarization of ASMCs resulting in vascular hyporeactivity (Brayden 2002; Chrissobolis and Sobey 2003). After pretreatment with MaxiK and K_ATP_ inhibitors, the improvement of vasoconstrictor hyporeactivity induced by DMY was significantly blocked, this indicated that MaxiK and K_ATP_ channels mediated the DMY-induced improvement of vasoconstrictor hyporeactivity. The compromised ability of an artery to constrict is likely to be caused by the defective function of the potassium channel in blood vessels, and it may be due to a change in unitary conductance, or open probability of the channels, and a change in expression number (Karabacak et al. 2015). We detected the currents of MaxiK and K_ATP_ by using electrophysiological methods. DMY blunted the high sensitized MaxiK and K_ATP_ channels of ASMCs isolated from the thoracic aorta of LPS rats.

Vasoplegia is thought to be a key aspect in the pathogenesis of cardiovascular alterations during severe sepsis and a key factor responsible for the death of patients with septic shock, due to the persistent and irreversible hypotension (Lundy and Trzeciak 2009). Oxidative stress during severe sepsis is one of the important factors resulting in vascular hyporeactivity to vasoconstrictors (Gamcrlidze et al. 2015). Inactivation of α-adrenoceptors by peroxynitrite may be a possible mechanism of cardiovascular hyporeactivity to catecholamines and systemic hypotension in sepsis (Shintani et al. 1996). It has been demonstrated that peroxynitrite scavenging improves contractile responses in aorta and microvasculature and has protective effects from vascular dysfunction in the sepsis, which is consistent with our results. Furthermore, some researchers proved that over-activation of K_ATP_ channels also participated in the vascular hyporeactivity to vasoconstrictors in sepsis (Rodrigo and Standen 2005; Sordi et al. 2011). Normally channel opening at the plasma membrane promotes K^+^ loss from the cell and maintenance of membrane resting potential. In vascular smooth muscle, potassium channels are extensively regulated by signalling pathways and cause vasodilation, contributing to both resting blood flow and vasodilator-induced increases in flow. Excessive activation of K^+^ channels on VSMCs membranes leads to membrane hyperpolarization, and the inhibition of Ca^2+^ entry through voltage-gated Ca^2+^ channels, thereby inducing cell relaxation, vasodilatation, and eventually resulting in hypotension and vascular hyporeactivity.

The relationship between potassium channels and oxidative stress has been established. Potassium channels can be over-activated by hypoxia, acidosis, hyperlactatemia, NO and peroxynitrite. Soh et al. (2001) reported that in neonatal rat hippocampal neurons, the reducing reagent glutathione (GSH) increased BK channel activity, whereas it’s oxidized form (GSSG) had the opposite effect. This suggests a redox modulatory mechanism when GSSG was applied to the intracellular side of the cell membrane. However, Zhang and Horrigan (2005) reported that after intracellular application of the oxidizing agent DTNB, there is an increase in open times and decrease in closing times of BK channels from adult native hippocampal CA1 pyramidal neurons. On the other hand, GSH had no apparent effect on BK channel activity.

The present study has some limitations. First, our experiments were performed in rats, and we did not have clinical data to support our conclusion. Second, considering its close relationship with blood pressure regulation, mesenteric artery strips, instead of thoracic aortic rings, it should be used to explore the vascular hyporeactivity of LPS and LPS + DMY rats. Finally, it would be desirable to silence or overexpress the MaxiK and K_ATP_ genes in order to explore the reasons for channel sensitization. A definitive causal relationship between MaxiK, K_ATP_ and DMY-induced improvement of vascular hyporeactivity could be drawn if the DMY-induced improvement of vascular hyporesponsiveness could be obviously inhibited after MaxiK and K_ATP_ channels expression changed.

In summary, our results indicate that the impaired contractility of ASMCs in experimental sepsis could be ameliorated by DMY administration. Such an effect is mediated by the normalization of the over-excited MaxiK and K_ATP_ channels, possibly via the inhibition of oxidative stress.

## References

[CIT0001] Brayden JE 2002 Functional roles of K_ATP_ channels in vascular smooth muscle. Clin Exp Pharmacol Physiol. 29:312–316. 11985542 10.1046/j.1440-1681.2002.03650.x

[CIT0002] Chrissobolis S, Sobey CG 2003 Inwardly rectifying potassium channels in the regulation of vascular tone. Curr Drug Targets. 4:281–289. 12699348 10.2174/1389450033491046

[CIT0003] Donaldson LL, Myers AK 1996 Effect of pharmacological agonists on contractile responses in aortic rings derived from endotoxaemic rats. J Vet Pharmacol Ther. 19:389–396. 8905574 10.1111/j.1365-2885.1996.tb00069.x

[CIT0004] Dopico AM, Walsh JV Jr, Singer JJ 2002 Natural bile acids and synthetic analogues modulate large conductance Ca^2+^-activated K^+^ (BKCa) channel activity in smooth muscle cells. J Gen Physiol. 119:251–273. 11865021 10.1085/jgp.20028537 PMC2217287

[CIT0005] Gamcrlidze MM, Intskirveli NA, Vardosanidze KD, Chikhladze Kh E, Goliadze L, Ratiani LR 2015 Vasoplegia in septic shock (review). Georgian Med News. 239:56–62. 25802451

[CIT0006] Hou XL, Tong Q, Wang WQ, Shi CY, Xiong W, Chen J, Liu X, Fang JG 2015 Suppression of inflammatory responses by dihydromyricetin, a flavonoid from *Ampelopsis grossedentata*, via inhibiting the activation of NF-κB and MAPK signaling pathways. J Nat Prod. 78:1689–1696. 26171689 10.1021/acs.jnatprod.5b00275

[CIT0007] Huang L, Liu J, Yu X, Shi L, Xiao H, Huang Y 2016 Drug-drug interactions between moxifloxacin and rifampicin based on pharmacokinetics *in vivo* in rats. Biomed Chromatogr. 30:1591–1598. 27028459 10.1002/bmc.3726

[CIT0008] Johnson D, Mayers I 2001 Multiple organ dysfunction syndrome: a narrative review. Can J Anaesth. 48:502–509. 11394523 10.1007/BF03028318

[CIT0009] Karabacak K, Kaya E, Ulusoy KG, Seyrek M, Kurtoglu M, Doganci S, Yildirim V, Yildiz O, Demirkilic U 2015 Effects of taurine on contractions of human internal mammary artery: a potassium channel opening action. Eur Rev Med Pharmacol Sci. 19:1498–1504. 25967726

[CIT0010] Koide M, Syed AU, Braas KM, May V, Wellman GC 2014 Pituitary adenylate cyclase activating polypeptide (PACAP) dilates cerebellar arteries through activation of large-conductance Ca^2+^-activated (BK) and ATP-sensitive (K_ATP_) K^+^ channels. J Mol Neurosci. 54:443–450. 24744252 10.1007/s12031-014-0301-z PMC4201911

[CIT0011] Kou X, Shen K, An Y, Qi S, Dai WX, Yin Z 2012 Ampelopsin inhibits H_2_O_2_-induced apoptosis by ERK and Akt signaling pathways and up-regulation of heme oxygenase-1. Phytother Res. 26:988–994. 22144097 10.1002/ptr.3671

[CIT0012] Leone M, Martin C 2008 Vasopressor use in septic shock: an update. Curr Opin Anaesthesiol. 21:141–147. 18443479 10.1097/ACO.0b013e3282f46d20

[CIT0013] Liu L, Yin X, Wang X, Li X 2017 Determination of dihydromyricetin in rat plasma by LC-MS/MS and its application to a pharmacokinetic study. Pharm Biol. 55:657–662. 27951743 10.1080/13880209.2016.1266669 PMC6130699

[CIT0014] Lundy DJ, Trzeciak S 2009 Microcirculatory dysfunction in sepsis. Crit Care Clin. 25:721–731. 19892249 10.1016/j.ccc.2009.06.002

[CIT0015] Pang R, Tao JY, Zhang SL, Chen KL, Zhao L, Yue X, Wang YF, Ye P, Zhu Y, Wu JG 2011 Ethanol extract from *Ampelopsis sinica* root exerts anti-hepatitis B virus activity via inhibition of p53 pathway *in vitro*. Evid Based Complement Alternat Med. 2011:939205. 21738555 10.1093/ecam/neq011 PMC3130517

[CIT0016] Rodrigo GC, Standen NB 2005 ATP-sensitive potassium channels. Curr Pharm Des. 11:1915–1940. 15974968 10.2174/1381612054021015

[CIT0017] Shi WW, Yang Y, Shi Y, Jiang C 2012 K_ATP_ channel action in vascular tone regulation: from genetics to diseases. Sheng Li Xue Bao. 64:1–13. 22348955 PMC4132831

[CIT0018] Shintani F, Kinoshita T, Kanba S, Ishikawa T, Suzuki E, Sasakawa N, Kato R, Asai M, Nakaki T 1996 Bioactive 6-nitronorepinephrine identified in mammalian brain. J Biol Chem. 271:13561–13565. 8662880 10.1074/jbc.271.23.13561

[CIT0019] Soh H, Jung W, Uhm DY, Chung S 2001 Modulation of large conductance calcium-activated potassium channels from rat hippocampal neurons by glutathione. Neurosci Lett. 298:115–118. 11163291 10.1016/s0304-3940(00)01737-7

[CIT0020] Sordi R, Fernandes D, Heckert BT, Assreuy J 2011 Early potassium channel blockade improves sepsis-induced organ damage and cardiovascular dysfunction. Br J Pharmacol. 163:1289–1301. 21410460 10.1111/j.1476-5381.2011.01324.x PMC3144541

[CIT0021] Spradley FT, Ho DH, Kang KT, Pollock DM, Pollock JS 2012 Changing standard chow diet promotes vascular NOS dysfunction in Dahl S rats. Am J Physiol Regul Integr Comp Physiol. 302:R150–R158. 22031779 10.1152/ajpregu.00482.2011 PMC3349380

[CIT0022] Strunk V, Hahnenkamp K, Schneuing M, Fischer LG, Rich GF 2001 Selective iNOS inhibition prevents hypotension in septic rats while preserving endothelium-dependent vasodilation. Anesth Analg. 92:681–687. 11226101 10.1097/00000539-200103000-00025

[CIT0023] Szabo C, Salzman AL, Ischiropoulos H 1995 Endotoxin triggers the expression of an inducible isoform of nitric oxide synthase and the formation of peroxynitrite in the rat aorta *in vivo*. FEBS Lett. 363:235–238. 7537701 10.1016/0014-5793(95)00322-z

[CIT0024] Won JH, Im HT, Kim YH, Yun KJ, Park HJ, Choi JW, Lee KT 2006 Anti-inflammatory effect of buddlejasaponin IV through the inhibition of iNOS and COX-2 expression in RAW 264.7 macrophages via the NF-kappaB inactivation. Br J Pharmacol. 148:216–225. 16520738 10.1038/sj.bjp.0706718 PMC1617058

[CIT0025] Wu F, Wilson JX, Tyml K 2004 Ascorbate protects against impaired arteriolar constriction in sepsis by inhibiting inducible nitric oxide synthase expression. Free Radic Biol Med. 37:1282–1289. 15451067 10.1016/j.freeradbiomed.2004.06.025

[CIT0026] Xin ML, Ma YJ, Xu K, Chen MC 2012 Structure-activity relationship for dihydromyricetin as a new natural antioxidant in polymer. J Appl Polym Sci. 128:1436–1442.

[CIT0027] Zhang G, Horrigan FT 2005 Cysteine modification alters voltage- and Ca(2+)-dependent gating of large conductance (BK) potassium channels. J Gen Physiol. 125:213–236. 15684095 10.1085/jgp.200409149 PMC2217493

[CIT0028] Zhang YS, Ning ZX, Yang SZ, Wu H 2003 Antioxidation properties and mechanism of action of dihydromyricetin from *Ampelopsis grossedentata*. Yao Xue Xue Bao. 38:241–244. 12889119

